# Bugs and Hydrophobia

**Published:** 1891-11

**Authors:** 


### BUGS AND HYDROPHOBIA.

A man lives in Turkalin, in the province of Livonia, Russia, who can cure the worst cases of hydrophobia. His medicine for the disease consists of bugs of the size of a firefly, which he gathers in the month of May in sandy places. He keeps them in clean bottles until they become perfectly dry and shrivelled to the size of the small common fly. Only one dose of two such bugs kneaded in bread is sufficient to cure the worst case of hydrophobia. When the patient takes the dose his temperature begins to rise. In about three hours he has a fit of raving madness, after which he falls asleep. When he awakes his disease is gone.

Emerson says: "The fiend that torments man is his love of the perfect," and speaking of man's insatiable desire for "more," Carlyle says: "No sooner is your ocean filled, than he grumbles that it might have been of better vintage. Try him with half of a universe, of an Omnipotence, he sets to quarreling with the proprietor of the other half, and declares himself the most maltreated of men."

